# Supplementary material for: TLR3 forms a laterally aligned multimeric complex along double-stranded RNA for efficient signal transduction
Source: Nat Commun. 2023 Jan 11;14:164. doi: 10.1038/s41467-023-35844-2 (PMC9834221; doi:10.1038/s41467-023-35844-2)
Supplement: Supplementary file 1 — Supplementary Information [file 41467_2023_35844_MOESM1_ESM.pdf]

# Supplementary Information

## TLR3 forms a laterally aligned multimeric complex along double-stranded RNA for efficient signal transduction


**Authors**

Kentaro Sakaniwa[1], Akiko Fujimura[1], Takuma Shibata[2], Hideki Shigematsu[3], Toru Ekimoto[4], Masaki Yamamoto[3], Mitsunori Ikeguchi[4], Kensuke Miyake[2], Umeharu Ohto[1]* and Toshiyuki Shimizu[1]*

***Corresponding author:**

Umeharu Ohto (U.O.)  E-mail: umeji@mol.f.u-tokyo.ac.jp

Toshiyuki Shimizu (T.S.)  E-mail: shimizu@mol.f.u-tokyo.ac.jp


This file includes:
Supplementary Figure legends 1-7
Supplementary Table 1

**Supplementary Table 1 Cryo-EM data collection, refinement, and validation statistics**

| | Mouse TLR3 (26-705)-dsRNA (90 bp) (EMD-32599, PDB 7WM4) |
|---|---|
| **Data collection and processing** | |
| Magnification | 60,000 |
| Voltage (kV) | 300 |
| Electron exposure ($e^{-}/Å^2$) | 50 |
| Defocus range (μm) | -1.4 to -2.4 |
| Pixel size (Å) | 0.752 |
| Symmetry imposed | *C*2 |
| Initial particle images (no.) | 10,866,015 |
| Final particle images (no.) | 274,002 |
| Map resolution (Å) | 3.2 |
| FSC threshold | 0.143 |
| | |
| **Refinement** | |
| Model resolution (Å) | 3.2 |
| FSC threshold | 0.5 |
| Map sharpening *B* factor ($Å^2$) | 95.9 |
| Model composition | |
| Non-hydrogen atoms | 25,848 |
| Protein residues: TLR3 | 2,658 |
| Nucleotides: dsRNA | 162 |
| Glycans: NAG, BAM | 82 |
| *B* factors ($Å^2$) | |
| TLR3 | 208.28 |
| DsRNA | 229.64 |
| Glycans | 232.76 |
| R.m.s. deviations | |
| Bond lengths (Å) | 0.002 |
| Bond angles (°) | 0.524 |
| | |
| **Validation** | |
| MolProbity score | 1.85 |
| Clashscore | 9.68 |
| Rotamer outliers (%) | 0.00 |
| Ramachandran plot | |
| Favored (%) | 95.11 |
| Allowed (%) | 4.81 |
| Outliers (%) | 0.08 |

(a)

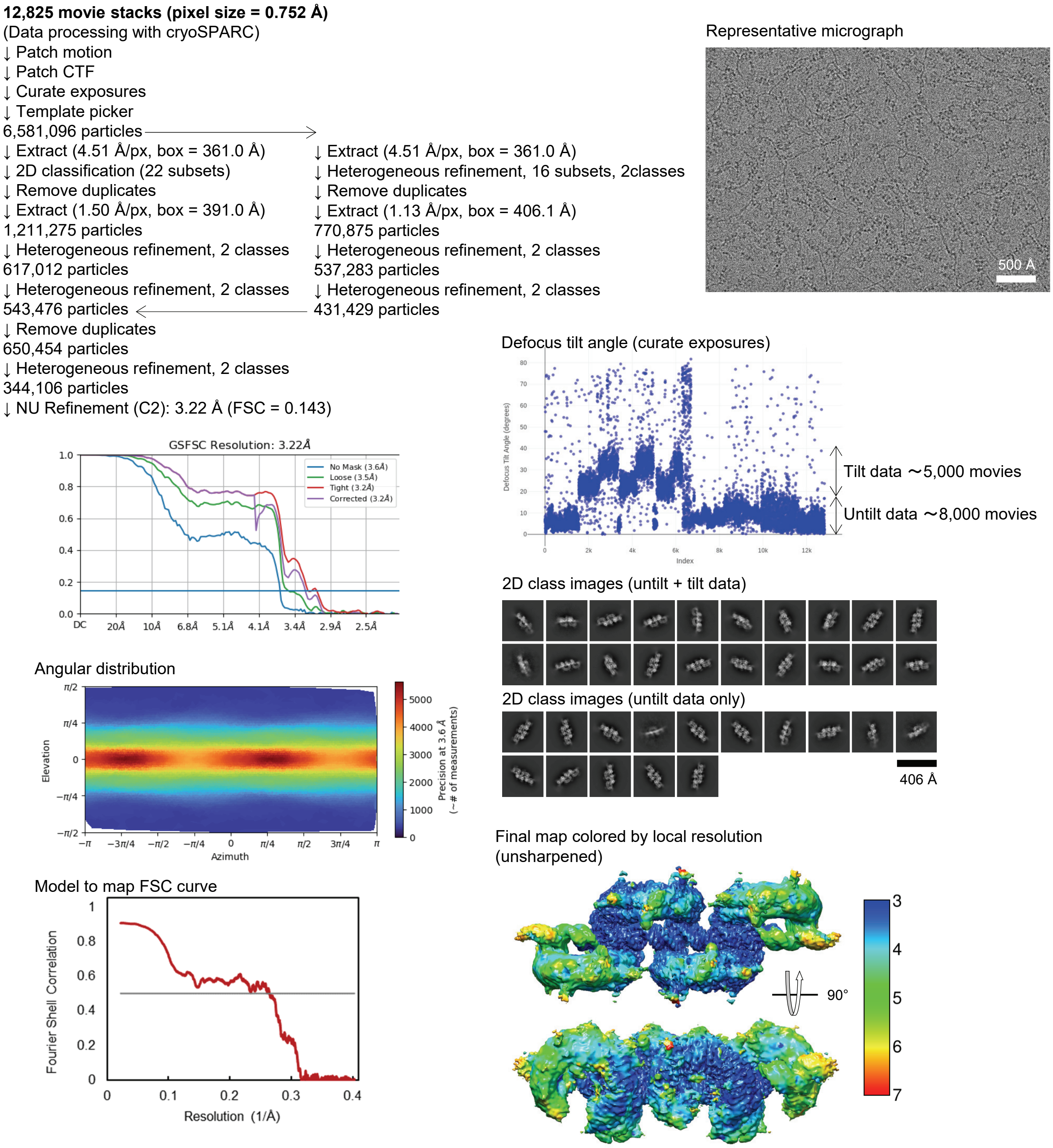
12,825 movie stacks (pixel size = 0.752 Å)
(Data processing with cryoSPARC)
↓ Patch motion
↓ Patch CTF
↓ Curate exposures
↓ Template picker
6,581,096 particles
↓ Extract (4.51 Å/px, box = 361.0 Å)
↓ 2D classification (22 subsets)
↓ Remove duplicates
↓ Extract (1.50 Å/px, box = 391.0 Å)
1,211,275 particles
↓ Heterogeneous refinement, 2 classes
617,012 particles
↓ Heterogeneous refinement, 2 classes
543,476 particles
↓ Remove duplicates
650,454 particles
↓ Heterogeneous refinement, 2 classes
344,106 particles
↓ NU Refinement (C2): 3.22 Å (FSC = 0.143)
↓ Extract (4.51 Å/px, box = 361.0 Å)
↓ Heterogeneous refinement, 16 subsets, 2classes
↓ Remove duplicates
↓ Extract (1.13 Å/px, box = 406.1 Å)
770,875 particles
↓ Heterogeneous refinement, 2 classes
537,283 particles
↓ Heterogeneous refinement, 2 classes
431,429 particles
Representative micrograph
500 Å
Defocus tilt angle (curate exposures)
Tilt data ～5,000 movies
Untilt data ～8,000 movies
GSFSC Resolution: 3.22Å
No Mask (3.6Å)
Loose (3.5Å)
Tight (3.2Å)
Corrected (3.2Å)
2D class images (untilt + tilt data)
2D class images (untilt data only)
406 Å
Angular distribution
Elevation
Azimuth
Precision at 3.6 Å (~ # of measurements)
Final map colored by local resolution (unsharpened)
90°
Model to map FSC curve
Fourier Shell Correlation
Resolution (1/Å)

(b)

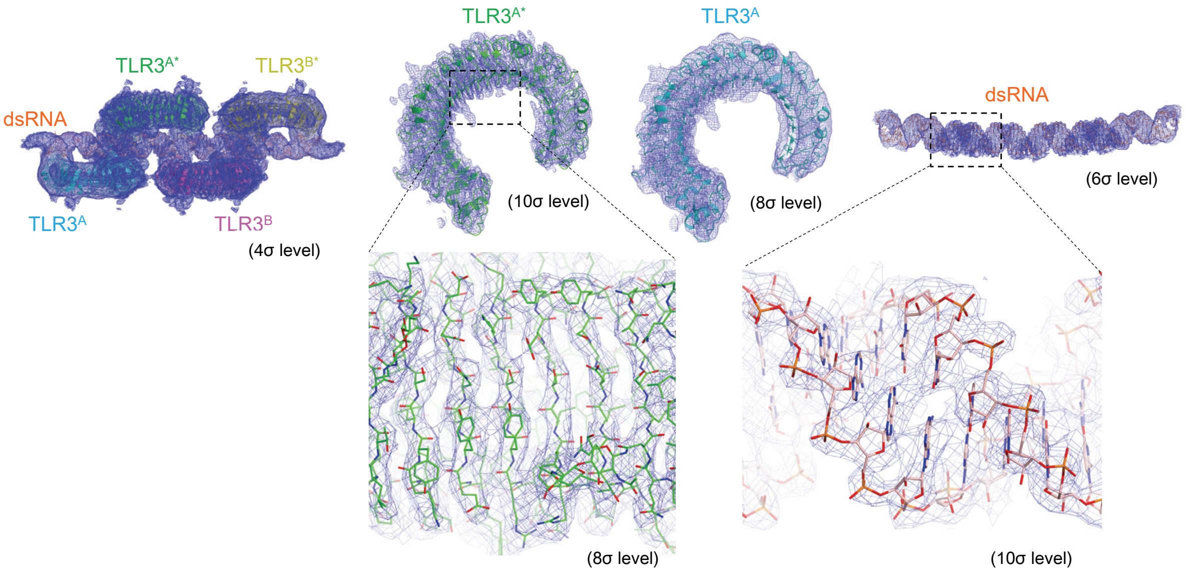
TLR3A*
TLR3B*
dsRNA
TLR3A
TLR3B
(4σ level)
TLR3A*
(10σ level)
TLR3A
(8σ level)
dsRNA
(6σ level)
(8σ level)
(10σ level)

**Supplementary Fig. 1 Cryo-EM data processing of the mouse TLR3-dsRNA (90 bp) complex.**

(a) Cryo-EM data analyses. Cryo-EM data processing workflow, a representative motion-corrected micrograph, defocus tilt angle, 2D classification images, gold-standard FSC curve (resolution cut-off at FSC = 0.143) and angular distribution of the final 3D reconstitution, the final 3D-map colored according to the local resolution, and model to map FSC curve are shown.

(b) Representative cryo-EM density maps of mouse TLR3-dsRNA (90 bp) complex. The map levels are indicated in each panel.

(a)

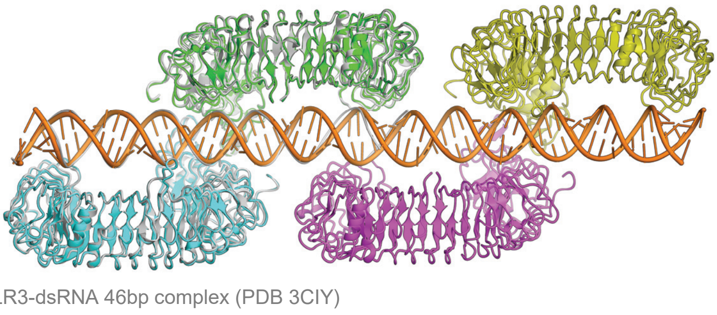


(b)

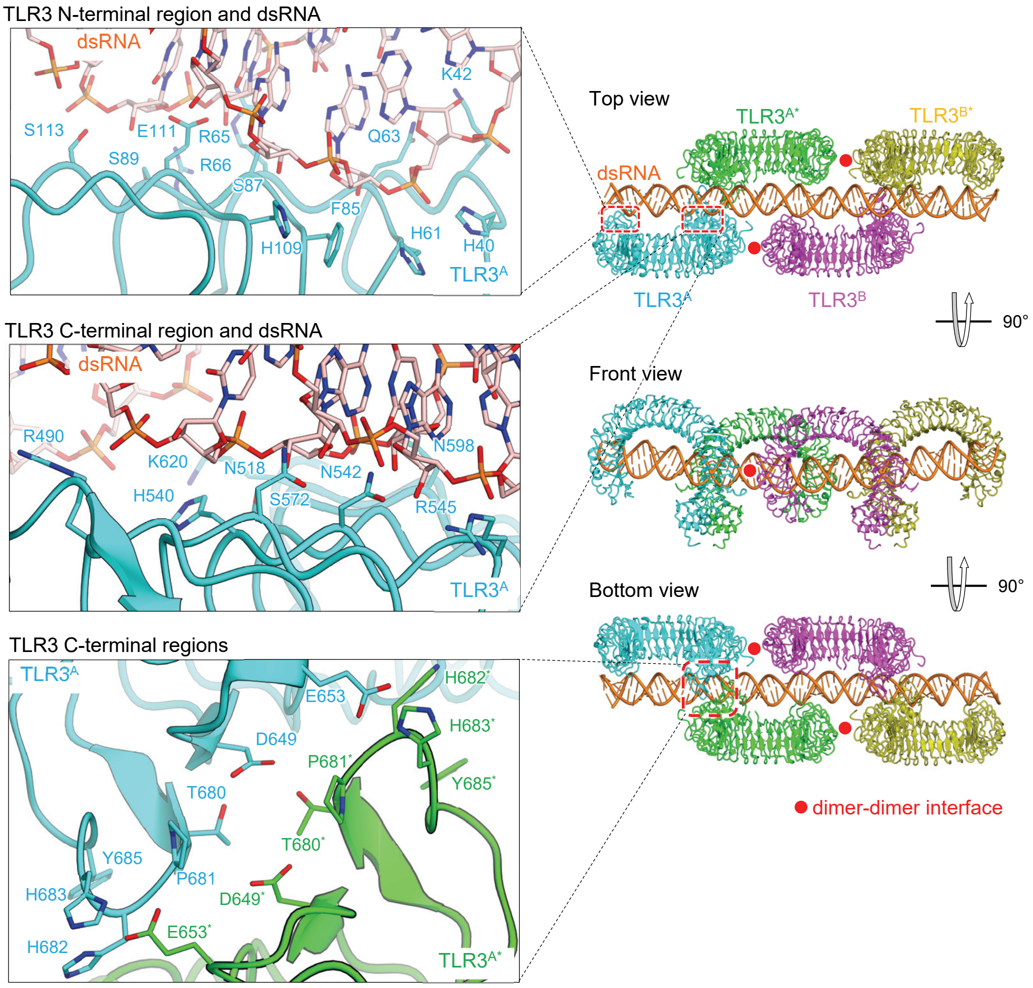


**Supplementary Fig. 2 TLR3 dimer unit in the mouse TLR3-dsRNA (90 bp) complex.**

(a) Structural comparison of the TLR3 dimer unit between the mouse TLR3-dsRNA (90 bp) complex (this study) and mouse TLR3-dsRNA (46 bp) complex colored gray (PDB 3CIY).

(b) The magnified views of the TLR3-dsRNA interfaces (left top and middle) and TLR3-TLR3* interface (left bottom) in the mouse TLR3-dsRNA (90 bp) complex, indicated by the dashed rectangles in the right panel. The residues involved in the TLR3-dsRNA or TLR3-TLR3* interactions are shown with sticks. The dimer-dimer interfaces are indicated by the red circle.

| dsRNA (fold) | 12.9 | 4.3 | 1.4 | 0.5 |
|---|---|---|---|---|
| tetramer | 1778 | 1468 | 3257 | 6642 |
| dimer | 12570 | 14174 | 14629 | 3677 |
| dimer + dimer | N.D. | N.D. | N.D. | 494 |
| tetramer + dimer | N.D. | N.D. | N.D. | 310 |
| tetramer (%) | 12.4 | 9.4 | 18.2 | 58.3 |

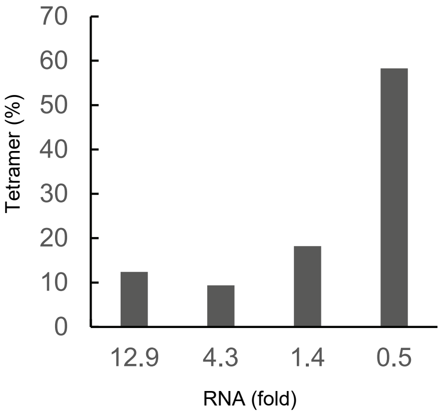


**Supplementary Fig. 3 Tetramer formation of TLR3 in the presence of different concentrations of dsRNA.**

The cryo-EM 2D class-averaged analyses were performed using WT TLR3 with different concentrations of 90 bp dsRNA.

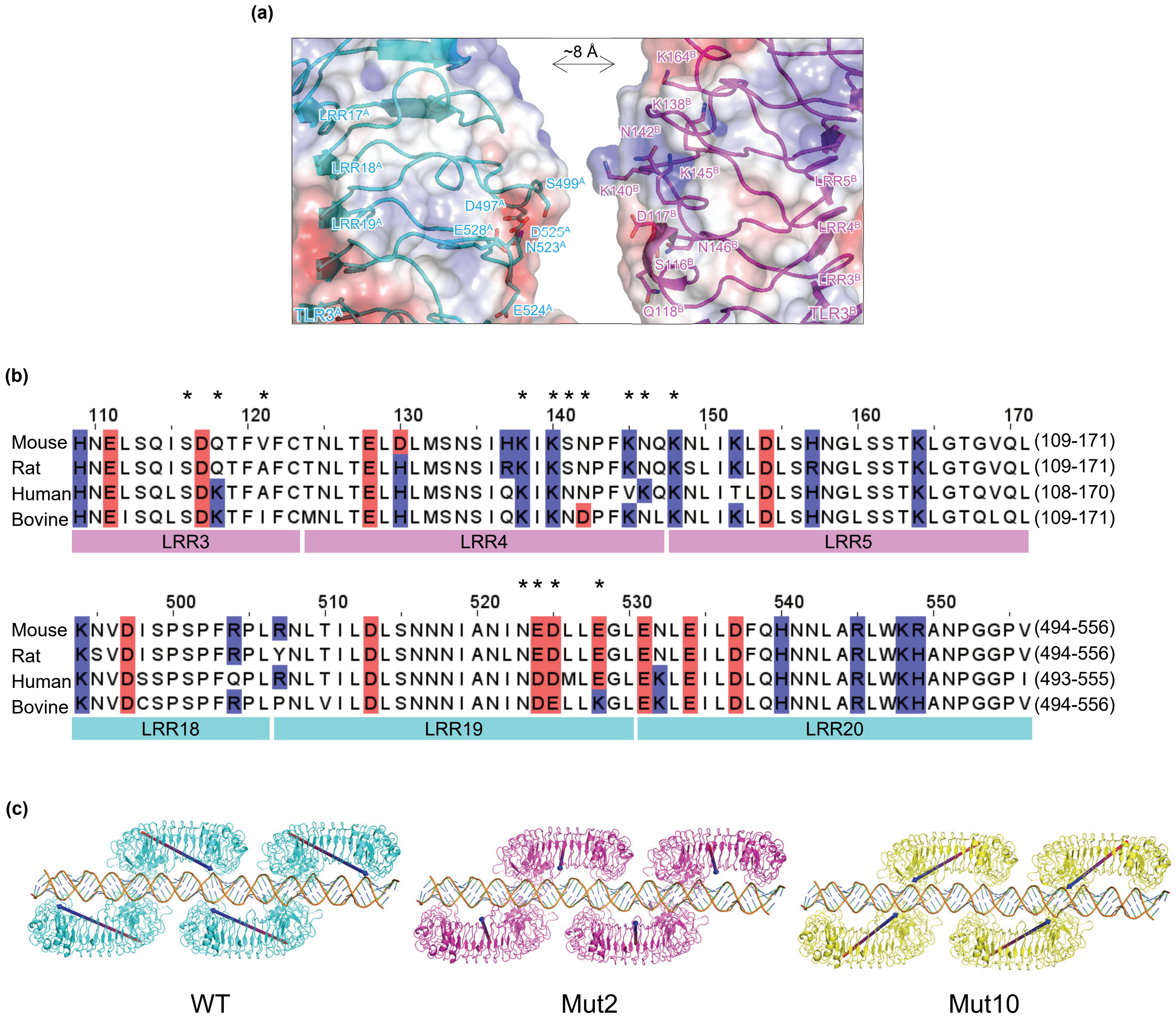


**Supplementary Fig. 4 Multiple sequence alignment of TLR3.**

(a) The close-up view of the dimer-dimer interface with the transparent electrostatic surface potentials map.

(b) Multiple sequence alignment of the mouse, rat, human, and bovine TLR3. The sequences of LRR3 to LRR5 (top) and LRR18 to LRR20 (bottom) are shown. The positively or negatively charged residues are highlighted with blue or red, respectively. The residues to which the mutations were introduced in this study are marked with asterisks (*).

(c) Dipole moment of WT or mutant TLR3 (Mut2, Mut10). Dipole moment is shown by the arrow.

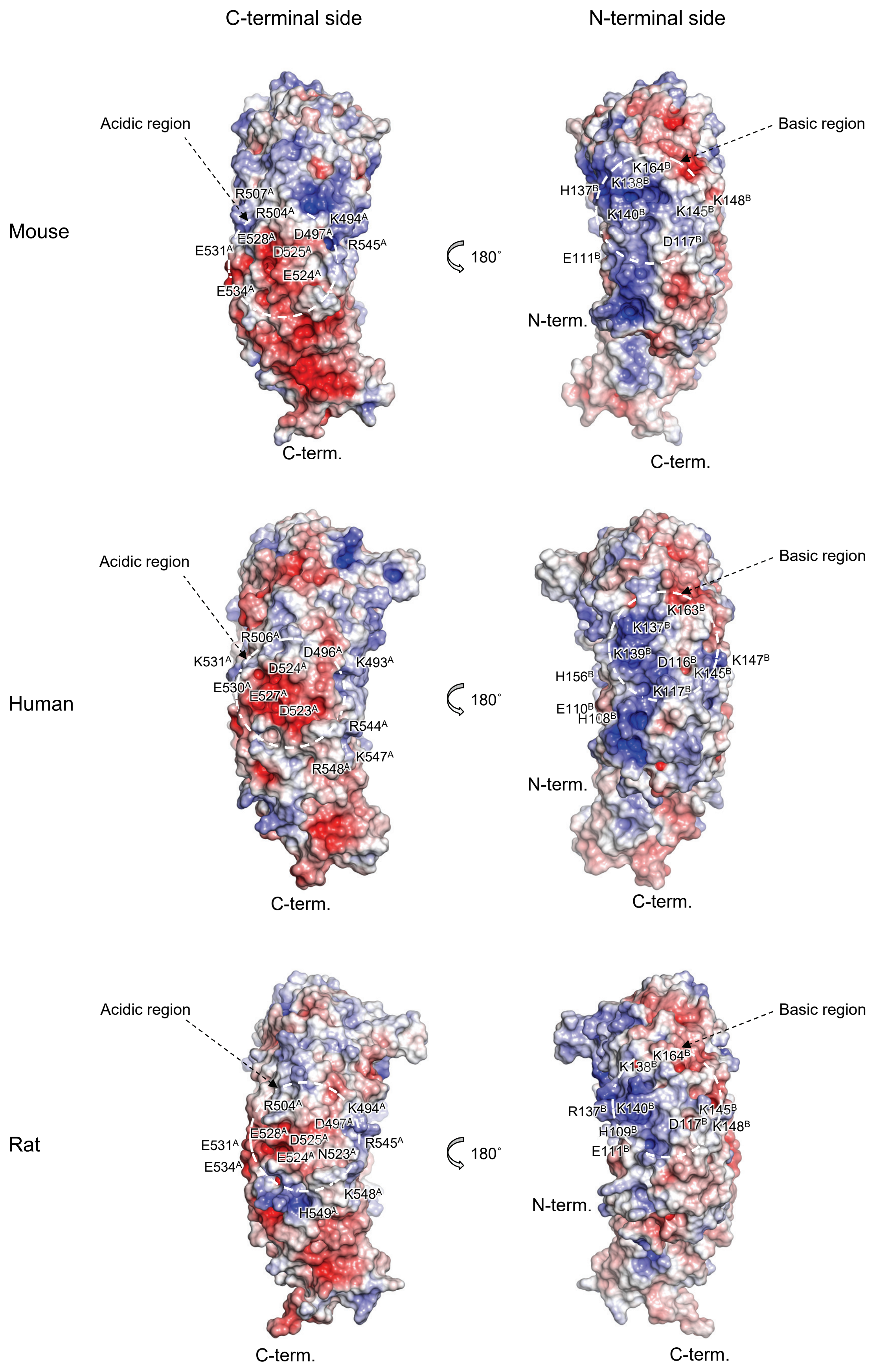


**Supplementary Fig. 5 The electrostatic surface potentials of TLR3.**

The electrostatic surface potentials of the convex surfaces of the C-terminal regions (left) and the N-terminal regions (right) for the mouse (PDB 3CIY) (top), human (PDB 2A0Z) (middle), and rat (Alphafold database) (bottom) TLR3 are shown. The positively- or negatively-charged regions near the dimer-dimer interface from this study are indicated by white dashed circles.

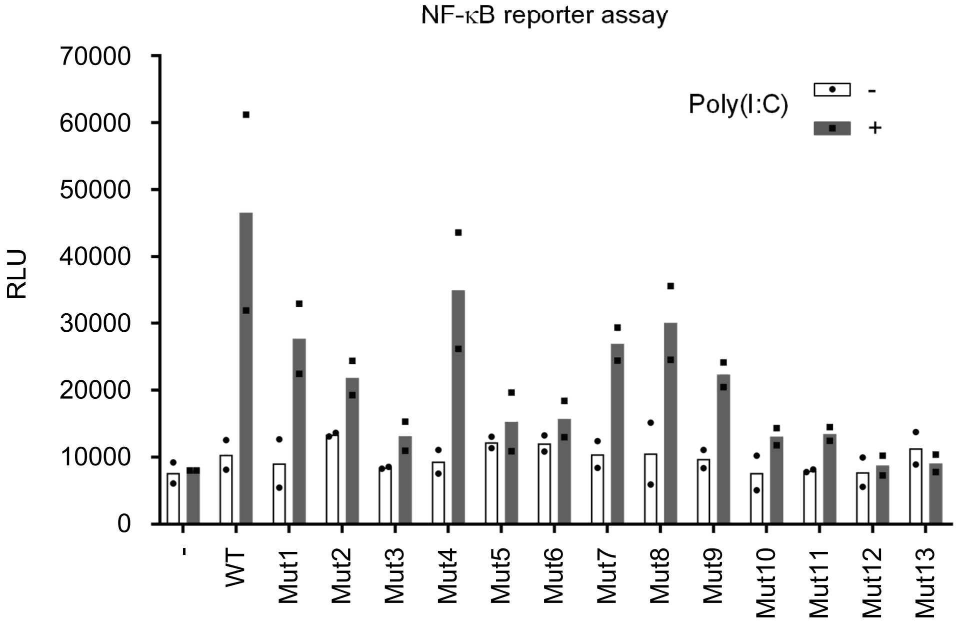


**Supplementary Fig. 6 NF-κB reporter assay of multimerization-defective TLR3 mutants.**

WT or mutant TLR3-expressing HEK293T cells were stimulated with 1 μg/mL poly (I:C) for 6 h and analyzed by NF-κB reporter assay. Bar plots are mean of n = 2 independent experiments.

(a)

**>80 movie stacks x2  from different grids (pixel size = 0.83 Å)**
Data processing with Relion
↓ Patch motion
↓ Patch CTF
↓ Autopicking with the references
↓ Extract (binning 6, box size = 480 Å)
↓ 2D classification
↓ Re-extract (binning 4, box size = 480 Å)
↓ 2D classification
↓ Re-extract (binning 3, box size = 480 Å)
↓ 2D classification (3 rounds)
↓ Particle selection to exclude duplicate particles
↓ Re-extract (binning 3, box size = 480 Å)
↓ 2D classification (30 classes) = Fig. 4a

2D references

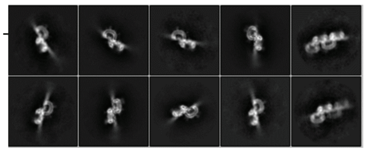

(b)

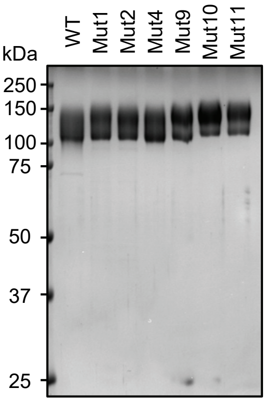


(c)

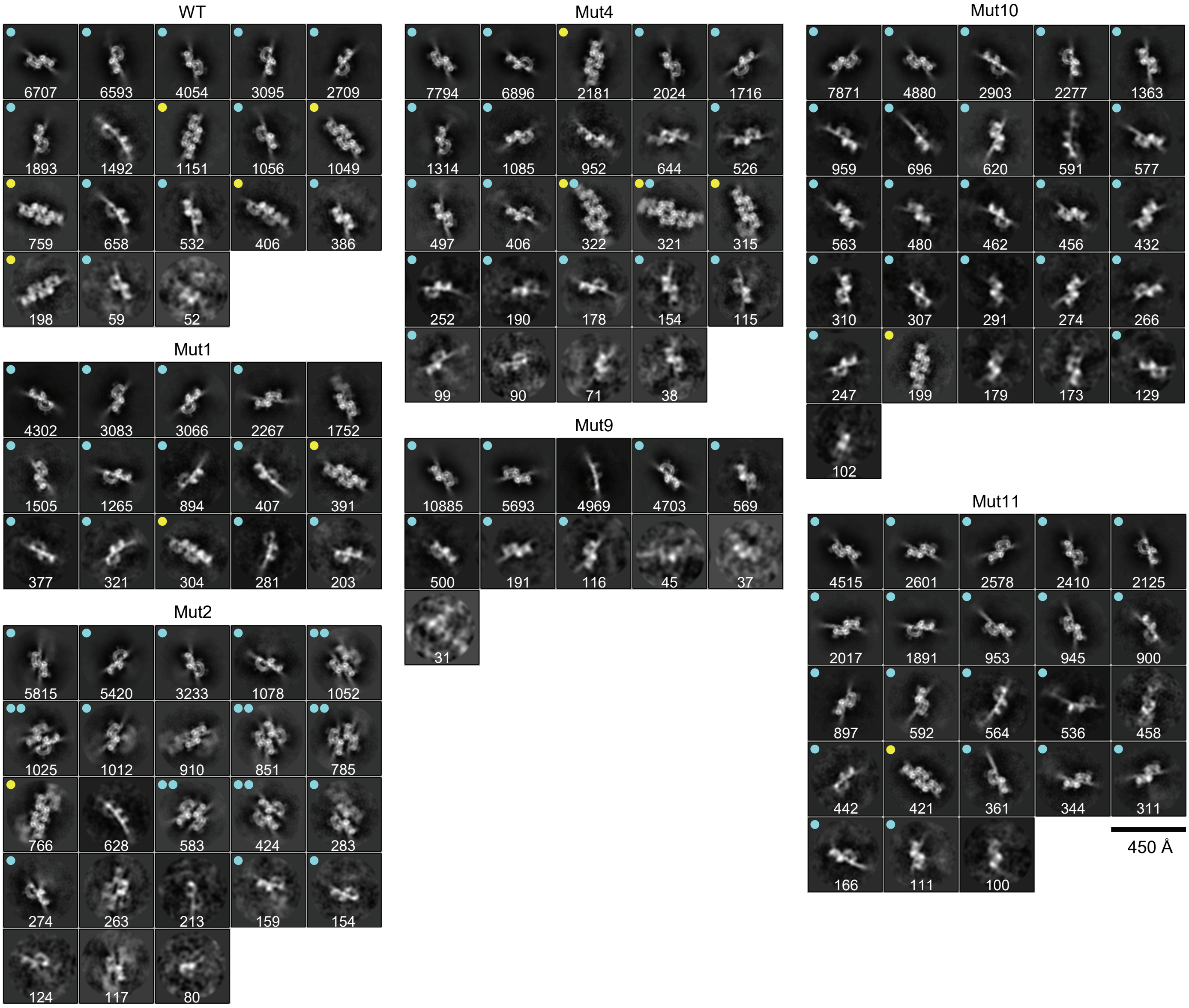


(d)

400 movie stacks (pixel size = 0.83 Å)
(data processing with cryoSPARC)
↓ Patch motion
↓ Patch CTF
↓ Template picker
449,865 particles
↓ Extract (1.245 Å/px, box = 249 Å)
↓ 2D classification
240,455 particles
↓ Heterogeneous refinement, 2 classes
122,126 particles
↓ NU Refinement (C2): 3.24 Å (FSC = 0.143)

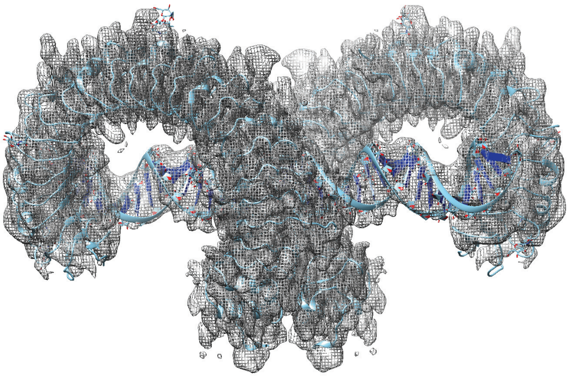

**Supplementary Fig. 7 Cryo-EM analyses of multimerization-defective TLR3 mutants.**

(a) The procedure of cryo-EM 2D analyses.

(b) The purified recombinant TLR3 mutants. The purified proteins were analysed by SDS-PAGE followed by Coomassie Brilliant Blue staining. Representative gel image in many trials is shown.

(c) Cryo-EM analyses of multimerization-defective TLR3 mutants in the presence of 90 bp dsRNA. 2D classification images in Fig. 3b are shown along with the number of particles in each class. The dimer and tetramer classes are indicated with light blue and yellow dot, respectively.

(d) Cryo-EM structure of TLR3 Mut10 (S116E, Q118E, K138E, K140E, N142E, K145E, N146E). Mouse TLR3-dsRNA 46bp complex (PDB 3CIY) is superposed onto the cryo-EM map of Mut10.
