# Supplementary material for: TLR3 forms a laterally aligned multimeric complex along double-stranded RNA for efficient signal transduction
Source: Nat Commun. 2023 Jan 11;14:164. doi: 10.1038/s41467-023-35844-2 (PMC9834221; doi:10.1038/s41467-023-35844-2)
Supplement: Supplementary file 3 — Reporting Summary [file 41467_2023_35844_MOESM3_ESM.pdf]

nature portfolio

Corresponding author(s): Toshiyuki Shimizu

Last updated by author(s): Dec 19, 2022

Reporting Summary

Nature Portfolio wishes to improve the reproducibility of the work that we publish. This form provides structure for consistency and transparency in reporting. For further information on Nature Portfolio policies, see our Editorial Policies and the Editorial Policy Checklist.

Statistics

For all statistical analyses, confirm that the following items are present in the figure legend, table legend, main text, or Methods section.

n/a	Confirmed	
☐	☒	The exact sample size (*n*) for each experimental group/condition, given as a discrete number and unit of measurement
☐	☒	A statement on whether measurements were taken from distinct samples or whether the same sample was measured repeatedly
☐	☒	The statistical test(s) used AND whether they are one- or two-sided<br>*Only common tests should be described solely by name; describe more complex techniques in the Methods section.*
☒	☐	A description of all covariates tested
☒	☐	A description of any assumptions or corrections, such as tests of normality and adjustment for multiple comparisons
☐	☒	A full description of the statistical parameters including central tendency (e.g. means) or other basic estimates (e.g. regression coefficient) AND variation (e.g. standard deviation) or associated estimates of uncertainty (e.g. confidence intervals)
☐	☒	For null hypothesis testing, the test statistic (e.g. F, t, r) with confidence intervals, effect sizes, degrees of freedom and P value noted<br>*Give P values as exact values whenever suitable.*
☒	☐	For Bayesian analysis, information on the choice of priors and Markov chain Monte Carlo settings
☒	☐	For hierarchical and complex designs, identification of the appropriate level for tests and full reporting of outcomes
☒	☐	Estimates of effect sizes (e.g. Cohen's d, Pearson's r), indicating how they were calculated

Our web collection on statistics for biologists contains articles on many of the points above.

Software and code

Policy information about availability of computer code

Data collection	Cryo-EM data were collected with the Serial EM software (3.9.0) and EPU software.
Data analysis	RELION (4.0), MotionCor2 (implemented in RELION), CTFFIND4 (implemented in RELION), cryoSPARC (3.3.2), Chimera (1.13.1), COOT (0.8.9.2), PHENIX (1.19.2-4158), PyMOL (1.8.x), CueMol2, APBS tools (PyMOL plugin).

For manuscripts utilizing custom algorithms or software that are central to the research but not yet described in published literature, software must be made available to editors and reviewers. We strongly encourage code deposition in a community repository (e.g. GitHub). See the Nature Portfolio guidelines for submitting code & software for further information.

Data

Policy information about availability of data

All manuscripts must include a data availability statement. This statement should provide the following information, where applicable:

- Accession codes, unique identifiers, or web links for publicly available datasets
- A description of any restrictions on data availability
- For clinical datasets or third party data, please ensure that the statement adheres to our policy

All data needed to evaluate the conclusions in the paper are present in the paper and/or Supplementary Figures and Table. Additional data and resources related to this paper may be requested from the authors. Cryo-EM maps and related structure coordinates of TLR3/dsRNA complex have been deposited in the EMDB and Protein Data Bank (PDB) under accession codes EMD-32599 [https://www.ebi.ac.uk/pdbe/emdb/entry/EMD-32599] and PDB: 7WM4 [http://doi.org/10.2210/pdb7WM4/pdb] respectively. For referenced structures, structure of TLR3/46bp dsRNA complex under accession codes PDB: 3CIY [http://doi.org/10.2210/pdb3CIY/pdb]. The source data underlying Figures 3b, 3c, 3e and Supplementary Figure 3, 6, 7b are provided as a Source Data file.

Field-specific reporting

Please select the one below that is the best fit for your research. If you are not sure, read the appropriate sections before making your selection.

☒ Life sciences ☐ Behavioural & social sciences ☐ Ecological, evolutionary & environmental sciences

For a reference copy of the document with all sections, see nature.com/documents/nr-reporting-summary-flat.pdf

Life sciences study design

All studies must disclose on these points even when the disclosure is negative.

Sample size	No statistical method was used to predetermine the sample size. Cryo-EM sample size were determined by the availability of microscope time and the number and quality of particles to obtain the reported images and structures. The sample size of each dataset is indicated in Method section and the image-processing procedures in the Supplementary Figure. Sample size of the cell-based assays was determined according to the conventions in the field.
Data exclusions	Cryo-EM images were excluded based on CTF max resolution parameters. Picked particles were excluded after 2D and 3D classification procedures using cryoSPARC and RELION software. Bad class averages were excluded manually based on general appearance, estimated resolutions and map quality. These procedures are general practice in the field of cryo-EM single particle analysis.
Replication	Recombinant protein purifications were performed several times. ISRE reporter, pull-down and NF-[illegible]B reporter assays were conducted at least two independent times and the replicates showed similar results.
Randomization	Randomization was performed when calculating Fourier-shell correlation of half maps. For other experiments, randomization was not applied. The statistical consideration is not relevant to these experiment because of the nature of biochemical and structural experiment performed in this work.
Blinding	Blinding was not relevant for cryo-EM data processing. Blinding is not technically or practically feasible for the biochemical experiments in this study.

Reporting for specific materials, systems and methods

We require information from authors about some types of materials, experimental systems and methods used in many studies. Here, indicate whether each material, system or method listed is relevant to your study. If you are not sure if a list item applies to your research, read the appropriate section before selecting a response.

Materials & experimental systems

n/a	Involved in the study
☐	☒ Antibodies
☐	☒ Eukaryotic cell lines
☒	☐ Palaeontology and archaeology
☒	☐ Animals and other organisms
☒	☐ Human research participants
☒	☐ Clinical data
☒	☐ Dual use research of concern

Methods

n/a	Involved in the study
☒	☐ ChIP-seq
☒	☐ Flow cytometry
☒	☐ MRI-based neuroimaging

Antibodies

Antibodies used	anti-DDDDK tag antibody: Medical & Biological Laboratories #M185-3L (Lot 015)<br>anti-βActin antibody: Santa Cruz Biotechnology, Inc. #sc-47778 (Lot J0421)<br>anti-mouse-HRP secondary antibody: Abcam #ab6728
Validation	All antibodies were validated by the respective manufacturers.<br>Anti-DDDDK tag antibody was validated through western blotting of various DDDDK-tagged proteins. Anti-βActin antibody was validated Western blotting of βActin-expressing cell lysate.

Eukaryotic cell lines

Policy information about cell lines

Cell line source(s)	HEK293T cell(Novus Biological, USA), Expi293F (ThermoFisher Scientific)
Authentication	For commercially available cell lines, no further cell line authentication was performed.

Mycoplasma contamination	No tested
Commonly misidentified lines (See ICLAC register)	No commonly misidentified cells were used in this study.
